# Efficacy and safety of peroxisome proliferator-activated receptor agonists for the treatment of primary biliary cholangitis: a meta-analysis of randomized controlled trials

**DOI:** 10.3389/fphar.2024.1432814

**Published:** 2024-07-22

**Authors:** Gang Tang, Jie Zhang, Linyu Zhang, Lingying Xia, Xiaojuan Tang, Rui Chen, Rongxing Zhou

**Affiliations:** ^1^ Division of Biliary Tract Surgery, Department of General Surgery, West China Hospital, Sichuan University, Chengdu, Sichuan, China; ^2^ Center for Translational Medicine, West China Second University Hospital, Sichuan University, Chengdu, Sichuan, China

**Keywords:** peroxisome proliferator-activated receptor agonist, primary biliary cholangitis, biochemical response, adverse events, meta-analysis

## Abstract

**Background:**

Peroxisome proliferator-activated receptor (PPAR) agonists are recognised as a promising treatment for primary biliary cholangitis (PBC). However, the effects and safety of these agonists on PBC remain unexplored. Our study aimed to investigate the efficacy and safety of PPAR agonists in treating PBC.

**Methods:**

We searched Cochrane Library, and Web of Science, PubMed, and Embase databases from inception to 15 March 2024 for randomised controlled studies (RCTs) that enrolled individuals with PBC treated with PPAR agonists compared with placebo. The primary outcomes were biochemical response and normalization of the alkaline phosphatase (ALP) level.

**Results:**

Eight RCTs involving 869 participants in total were included. The meta-analysis revealed that compared to placebo, PPAR agonists increased the rate of biochemical response (RR: 5.53; 95% CI: 3.79, 8.06) and normalization of the ALP level (RR: 17.18; 95% CI: 5.61, 52.61). In addition, PPAR agonists can also reduce alanine aminotransferase (ALT) (MD: −12.69 U/L; 95% CI: −18.03, −7.35), aspartate aminotransferase (AST) (MD: −4.18 U/L; 95% CI: −7.28, −1.08), ALP (MD: −142.95 U/L; 95% CI: −167.29, −118.60), γ-glutamyltransferase (GGT) (MD: −63.03 U/L; 95% CI: −92.08, −33.98), and total cholesterol (TC) levels (SMD: −0.71; 95% CI: −1.38, −0.04), and there was no significant difference in overall adverse reactions (RR: 0.99; 95% CI: 0.92, 1.05), serious adverse reactions (RR: 1.10; 95% CI: 0.70, 1.72) between the two groups.

**Conclusion:**

PPAR agonists are safe and well-tolerated in patients with PBC and are effective in improving the rate of biochemical response and related biomarkers.

## 1 Introduction

Primary biliary cholangitis (PBC) is an autoimmune mediated liver disease, characterized by interlobular bile duct destruction, hepatocellular toxicity endogenous bile acid retention and liver fibrosis, with an increasing prevalence worldwide (Montano-Loza and Corpechot, 2021; Jang et al., 2023; Kowdley et al., 2024). If poorly controlled, primary biliary cholangitis may progress to cirrhosis and liver failure (Levy et al., 2023; Hirschfield et al., 2024). The first line of treatment for PBC is ursodeoxycholic acid (UDCA). UDCA can effectively improve the level of liver biochemical indexes, delay the progression of disease, and prolong the survival without transplantation (Colapietro et al., 2023). However, up to 40% of patients have an inadequate response to UDCA, with elevated alkaline phosphatase levels and or bilirubin levels (Hirschfield et al., 2024). Importantly, patients with an inadequate response to UDCA therapy had a significantly increased risk of progression to end-stage liver disease and death compared to UDCA therapy responders (Gazda et al., 2023). Obeticholic acid is a selective farnesoid X receptor agonist and is the only FDA-approved second-line treatment for PBC. However, nearly 50% of patients still respond inadequately to the combination of obeticholic acid and UDCA (Jones et al., 2017). In addition, obeticholic acid was associated with higher rates of pruritus and serious adverse events compared to placebo (Corpechot et al., 2018). Therefore, there is an urgent need to develop new therapeutic drugs.

In recent years, a number of new drugs have entered the research and development stage, showing good results in clinical trials. Among them, peroxisome proliferator-activated receptor (PPAR) agonists have attracted great attention (Colapietro et al., 2023). PPAR agonists are a class of nuclear receptors that play a crucial role in regulating lipid metabolism, glucose homeostasis, and inflammatory responses, making them a key molecular target for the treatment of cholestatic liver disease, such as PBC (Colapietro et al., 2023). In animal models, PPAR agonists can effectively relieve the degree of cholangitis in mice (Nozaki et al., 2013), improve the symptoms of intrahepatic cholestasis in mice and reduce cholestation-related dyslipidemia (Zhang et al., 2020). In addition, several recent clinical studies (Jones et al., 2017; Schattenberg et al., 2021; Vuppalanchi et al., 2022) have reported the results of Phase 2 clinical trials of PPAR agonists for the treatment of PBC, highlighting their potential as novel therapeutics for PBC. However, there is a lack of comprehensive and systematic analysis to summarize this evidence.

Therefore, we conducted this systematic review and meta-analysis to elucidate the effects and safety of PPAR agonists on PBC. We believe our findings would help provide a clearer understanding of the value and potential of PPAR agonists in the treatment of PBC.

## 2 Methods

### 2.1 Search strategy

The study was conducted following the Preferred Reporting Items for Systematic Reviews and Meta-Analyses (PRISMA) statement (Page et al., 2021) and have been prospectively registered in the PROSPERO database (CRD42024538227).

We searched Cochrane Library, Web of Science, PubMed, and Embase databases from inception to 15 March 2024. The full search strategy is listed in Table 1. In addition, we checked the reference lists of the identified articles and related reviews to further screen eligible studies. There were no language restrictions in the search.

**TABLE 1:** Electronic search strategy.

Database	Search term (published up to 15 March 2024)	Number
PubMed	(peroxisome proliferator-activated receptor agonist OR PPAR OR bezafibrate OR fenofibrate OR seladelpar OR MBX-8025 OR elafibranor OR saroglitazar) AND (primary biliary cholangitis OR primary biliary cirrhosis OR PBC) AND (randomised controlled trial OR controlled clinical trial OR randomised OR randomly OR RCT)	47
Embase	(peroxisome proliferator-activated receptor agonist OR PPAR OR bezafibrate OR fenofibrate OR seladelpar OR MBX-8025 OR elafibranor OR saroglitazar).af. AND (primary biliary cholangitis OR primary biliary cirrhosis OR PBC).af. AND (randomised controlled trial OR controlled clinical trial OR randomised OR randomly OR RCT).af	45
Cochrane Library Trials	(peroxisome proliferator-activated receptor agonist OR PPAR OR bezafibrate OR fenofibrate OR seladelpar OR MBX-8025 OR elafibranor OR saroglitazar) AND (primary biliary cholangitis OR primary biliary cirrhosis OR PBC) AND (randomised controlled trial OR controlled clinical trial OR randomised OR randomly OR RCT)	88
Web of Science	(TS=(peroxisome proliferator-activated receptor agonist OR PPAR OR bezafibrate OR fenofibrate OR seladelpar OR MBX-8025 OR elafibranor OR saroglitazar)) AND (TS=(primary biliary cholangitis OR primary biliary cirrhosis OR PBC)) AND (TS=(randomised controlled trial OR controlled clinical trial OR randomised OR randomly OR RCT))	141

### 2.2 Study selection

Studies included in this meta-analysis were chosen according to the patient, intervention, comparator, outcome, and study type (PICOS) criteria. (1) Patient: Adults with PBC; (2) Intervention: PPAR agonists; (3) Comparator: placebo; (4) Outcome: The primary outcomes were biochemical response (defined as an alkaline phosphatase (ALP) level <1.67 times the ULN, with a reduction of ≥15% from baseline, and total bilirubin at or below the ULN) and normalization of the ALP level. Secondary outcomes includedthe levels of ALP, alanine aminotransferase (ALT), aspartate aminotransferase (AST), triglyceride (TG), high density lipoprotein cholesterol (HDL), low density lipoprotein cholesterol (LDL), γ-glutamyltransferase (GGT), total bilirubin (TB), total cholesterol (TC), and adverse events; (5) Study type: randomized controlled trials (RCTs). Reviews, case reports, editorials, letters, animal studies, and studies without control groups were excluded. Two authors (GT and LZ) conducted the study selection independently, and any disagreements were resolved through discussion with the third author (XT).

### 2.3 Data extraction

Data from all eligible studies were independently extracted by two reviewers (GT and LZ) based on a previously established form, and any disagreements were resolved by discussion with a third-party independent reviewer (XT). The main fields to be extracted included the author name, year of publication, country in which the study was conducted, study design, study population (sample size, age, and sex) and outcomes. When data of interest in an article were unavailable, the corresponding author was contacted to obtain the necessary data.

### 2.4 Quality assessment

Two investigators evaluated the quality of all the studies using the Cochrane risk-of-bias tool 2: (1) randomization process, (2) deviations from intended interventions, (3) missing outcome data, (4) measurement of the outcome, (5) selection of the reported results, and (6) overall risk of bias. Any discrepancy was resolved through discussion and intervention by a third reviewer whenever necessary. The evidence was graded using the Grading of Recommendations Assessment, Development and Evaluation (GRADE) framework.

### 2.5 Statistical analysis

The meta-analysis was performed using the Review 5.3 (The Nordic Cochrane Centre, The Cochrane Collaboration 2014; Copenhagen, Denmark). Effect estimates are presented as the mean difference (MD) or standardized mean differences (SMD) for continuous outcomes, and the risk ratio (RR) for dichotomous outcomes, with 95% confidence intervals (CIs). Heterogeneity was assessed using measures I^2^ test. The random-effects model was used when there was significant heterogeneity with the I^2^ > 50%. Otherwise, the fixed-effects model was adopted (Alesi et al., 2023).To assess the robustness of the results, sensitivity analyses were performed using one-study excluding method. When a study includes multiple intervention groups, we combine data from multiple intervention groups into a single group to avoid including individuals from the placebo group multiple times in the analysis. For trials that did not report net changes but provided baseline and post-intervention data, we calculated the post-intervention parameters minus the baseline parameters as the net change. The following formula was used to calculate SDs of the mean changes: SD = square root [(SD_pretreatment_)^2^ + (SD_posttreatment_)^2^ - 2r × SD_pretreatment_ × SD_posttreatment_], where the correlation coefficient (r) = 0.5 (16). Statistical significance was set at *p* < 0.05.

## 3 Results

### 3.1 Literature retrieval

**FIGURE 1:**
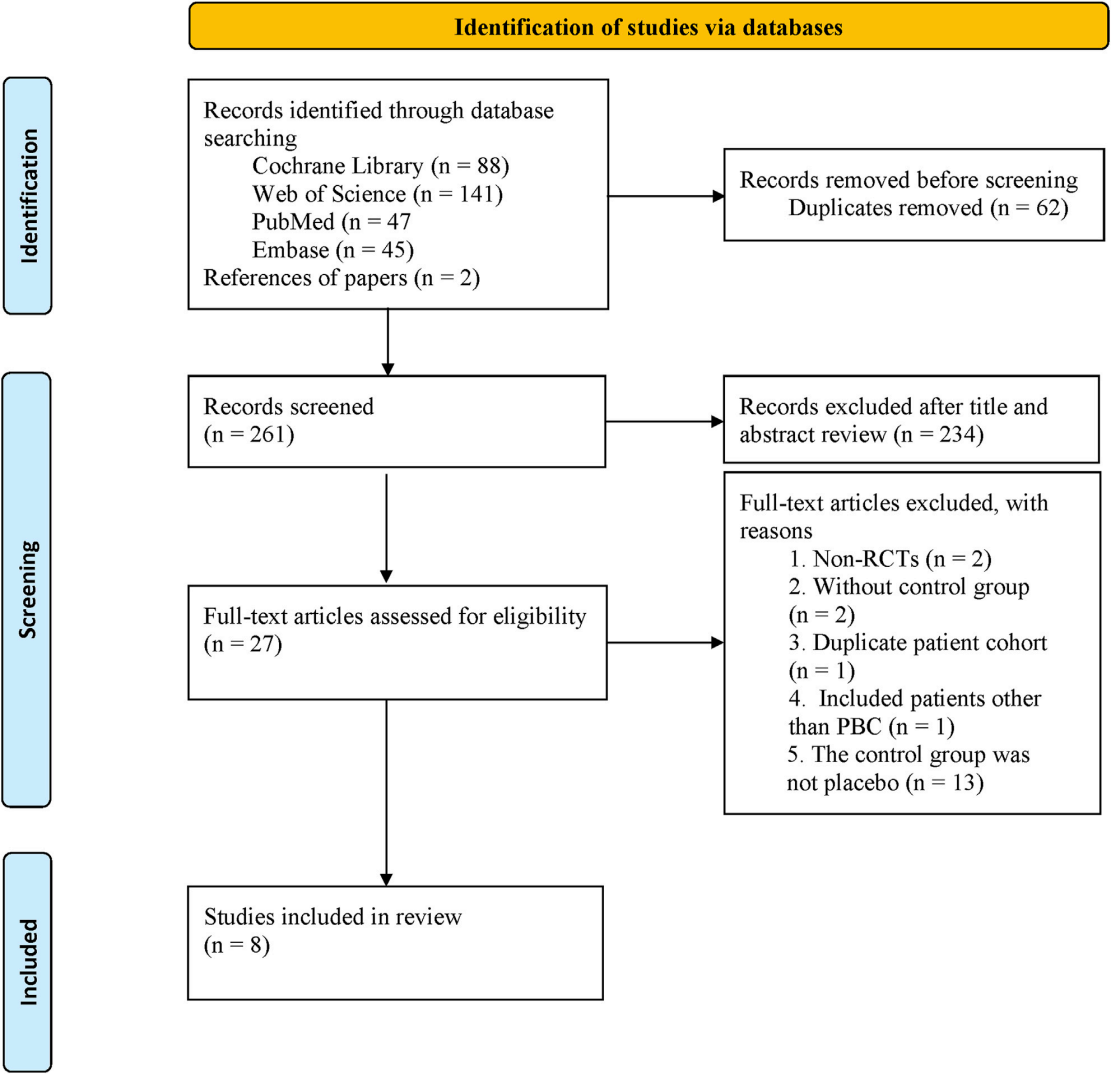
PRISMA flow diagram of the literature retrieval process.

We identified 323 studies from the initial search, leaving 261 studies after removing duplicates. After screening titles and abstracts, 27 potentially relevant studies were evaluated in full-text. Finally, 8 (Jones et al., 2017; Corpechot et al., 2018; Schattenberg et al., 2021; Hatami et al., 2022; Vuppalanchi et al., 2022; Hirschfield et al., 2023; Kowdley et al., 2024; Hirschfield et al., 2024) studies were included in our meta-analysis (Figure 1).

### 3.2 Study characteristics and quality assessment

Table 2 summarizes the primary characteristics of the included studies. The trials were published between 2017 and 2024 and included a total of 869 participants (561 in the PPAR agonists group and 308 in the placebo group). Of the eight studies we included, four (Jones et al., 2017; Schattenberg et al., 2021; Vuppalanchi et al., 2022; Hirschfield et al., 2023) involved multiple parallel experimental groups (one control group and multiple intervention groups). Three studies (Jones et al., 2017; Hirschfield et al., 2023; Hirschfield et al., 2024) evaluated the efficacy and safety of seladelpar, two studies (Schattenberg et al., 2021; Kowdley et al., 2024) elafibranor, one study (Corpechot et al., 2018) bezafibrate, one study (Vuppalanchi et al., 2022) saroglitazar, and one study (Hatami et al., 2022) fenofibrate. The number of participants per study ranged from 30 to 265, with intervention times ranging from 3 months to 24 months.

**TABLE 2:** Characteristics of all eligible studies.

First author, year	Country	Population	Sample size	Age	Gender (M/F)	Intervention description	Comparison description	Duration	Outcomes
Jones, 2017	29 centres in North America and Europe	PBC who were required to be on a stable and recommended dose of UDCA for the past 12 months and to have an ALP of at least 1·67 times the ULN	38	55.8 (9.2)	2 M/36	Seladelpar: 50 mg once-daily Seladelpar: 200 mg once-daily	Placebo	3 months	Number of patients with normalisation of ALP, ALP
Corpechot, 2018	21 centers in France	PBC who had had an inadequate response to UDCA alone	100	53 (10)	5 M/95	Bezafibrate: 400 mg once-daily	Placebo	24 months	Biochemical response, ALP, ALT, AST, TB, GGT, TC, LDL, HDL, adverse events
Schattenberg, 2021	21 centres in the US and Europe	PBC (All patients were treated with UDCA for at least 12 months and were at a stable dose for at least 6 months prior to randomization)	45	59.1 (8.15)	2 M/43	Elafibranor: 80 mg once-daily Elafibranor: 120 mg once-daily	Placebo	3 months	Biochemical response, ALP, ALT, AST, TB, GGT, TC, TG, LDL, HDL, adverse events
Vuppalanchi, 2021	10 centers in the United States of America	PBC with inadequate response to a year of UDCA therapy and ALP level of at least 1.67x the ULN at both screening visits 1 and 2 with <30% variance and total bilirubin less than or equal to 2x the ULN	37	57 (8.4)	1 M/37	Saroglitazar: 2 mg once-daily Saroglitazar: 4 mg once-daily	Placebo	4 months	Number of patients with normalisation of ALP, biochemical response, ALP, ALT, AST, TB, GGT, adverse events
Hatami, 2022	Iran	PBC	30	40.2 (9.2)	11 M/19	Fenofibrate: 200 mg once-daily	Placebo	6 months	ALP, ALT, AST
Hirschfield, 2023	111 sites in 21 countries	PBC (Patients must have been receiving a stable and recommended UDCA dose for the prior 12 months unless they were UDCA intolerant)	265	55.4 (9.0)	15 M/250	Seladelpar: 5 mg once-daily Seladelpar: 10 mg once-daily	Placebo	12 months	Number of patients with normalisation of ALP, biochemical response, ALP, ALT, AST, TB, GGT, TC, TG, LDL, HDL, adverse events
Hirschfield, 2024	90 sites in 24 countries	PBC treatment with UDCA for at least 12 months or a history of unacceptable side effects with UDCA	193	56.7 (9.7)	10 M/183	Seladelpar: 10 mg once-daily	Placebo	12 months	Number of patients with normalisation of ALP, biochemical response, ALP, ALT, AST, GGT, TC, TG, LDL, HDL, adverse events
Kowdley, 2024	82 sites in 14 countries	PBC who had an inadequate response to or unacceptable side effects with UDCA	161	57.1 (8.7)	7 M/154	Elafibranor: 80 mg once-daily	Placebo	13 months	Number of patients with normalisation of ALP, biochemical response, ALP, ALT, AST, GGT, TB, adverse events

ALP, alkaline phosphatase; ALT, alanine transaminase; AST, aspartate transaminase; F, female; GGT, γ-glutamyltransferase; HDL, high density lipoprotein; LDL, low density lipoprotein; M, male; PBC, primary biliary cholangitis; TB, total bilirubin; TC, total cholesterol; TG, triglyceride; UDCA, ursodeoxycholic acid.

### 3.3 Quality assessment

**FIGURE 2:**
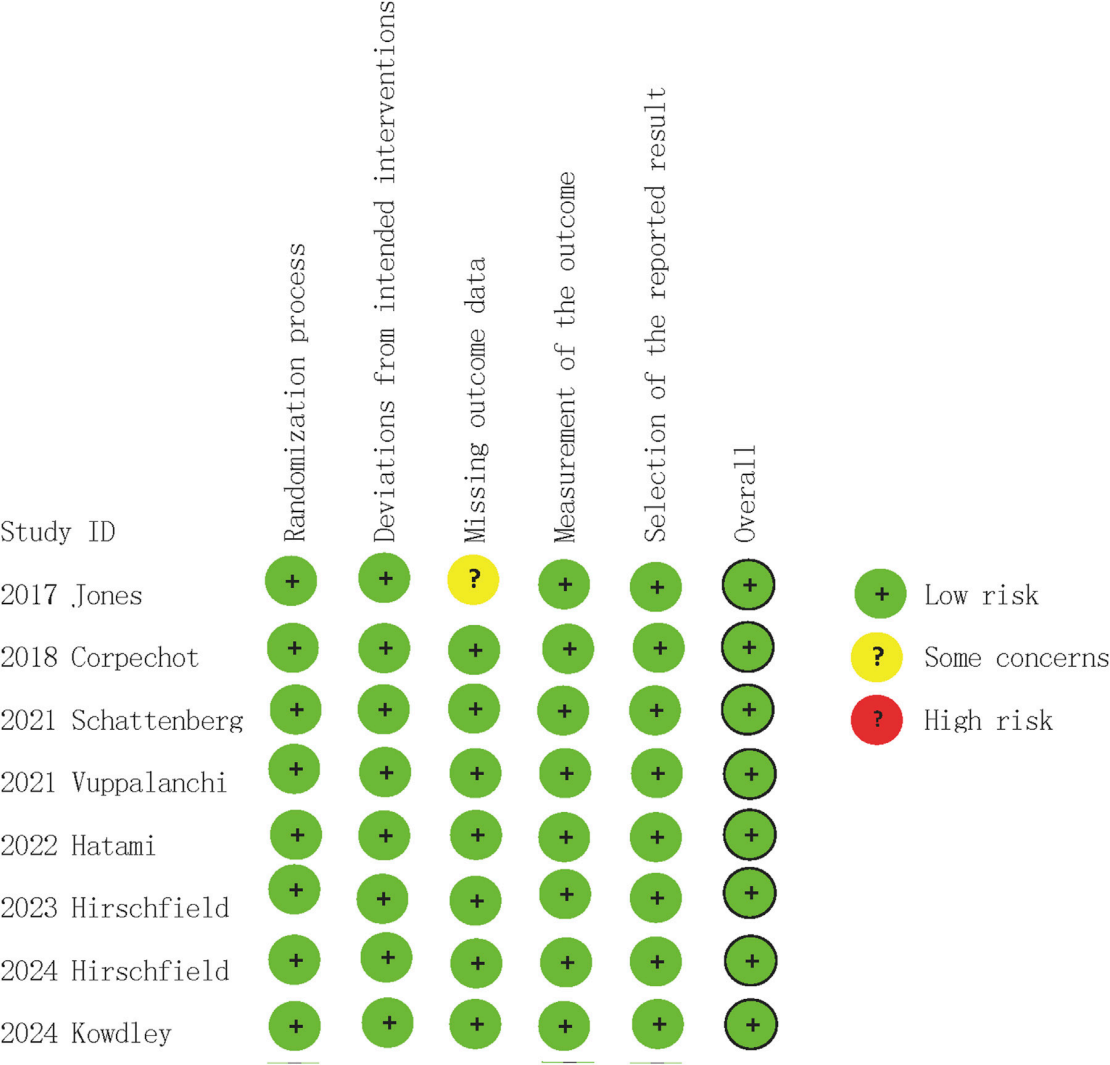
Risk of bias for each included study.

In quality assessment, eight studies (Jones et al., 2017; Corpechot et al., 2018; Schattenberg et al., 2021; Hatami et al., 2022; Vuppalanchi et al., 2022; Hirschfield et al., 2023; Kowdley et al., 2024; Hirschfield et al., 2024) were assessed as being of low risk of bias (Figure 2). All included studies were randomised and double-blind, with appropriate allocation concealment. In the GRADE assessments, the certainty of evidence in the reported outcomes mostly ranged from low to high, due to concerns regarding statistical heterogeneity (inconsistency) (Table 3).

**TABLE 3:** GRADE assessment for primary and secondary outcomes.

Certainty assessment							No of patients		Effect		Certainty	Importance
No of studies	Study design	Risk of bias	Inconsistency	Indirectness	Imprecision	Other considerations	PPAR agonist	Placebo	Relative (95% CI)	Absolute (95% CI)		
**Biochemical response**												
6	randomised trials	not serious	not serious	not serious	not serious	none	264/453 (58.3%)	24/249 (9.6%)	**RR 5.53** (3.79–8.06)	**437 more per 1,000** (from 269 more to 680 more)	⊕ ⊕ ⊕ ⊕ High	CRITICAL
**Normalization of the ALP level**												
6	randomised trials	not serious	not serious	not serious	not serious	none	88/373 (23.6%)	0/212 (0.0%)	**RR 17.18** (5.61–52.61)	**0 fewer per 1,000** (from 0 fewer to 0 fewer)	⊕ ⊕ ⊕ ⊕ High	CRITICAL
**ALP**												
8	randomised trials	not serious	serious^a^	not serious	not serious	none	560	308	-	MD **142.95 lower** (167.29 lower to 118.6 lower)	⊕ ⊕ ⊕ ○ Moderate	IMPORTANT
**ALT**												
7	randomised trials	not serious	serious^b^	not serious	not serious	none	535	295	-	MD **12.69 lower** (18.03 lower to 7.35 lower)	⊕ ⊕ ⊕ ○ Moderate	IMPORTANT
**AST**												
7	randomised trials	serious^c^	serious^d^	not serious	not serious	none	535	295	-	MD **4.18 lower** (7.28 lower to 1.08 lower)	⊕ ⊕ ○ ○ Low	IMPORTANT
**GGT**												
6	randomised trials	not serious	very serious^e^	not serious	not serious	none	520	280	-	MD **63.03 lower** (92.08 lower to 33.98 higher)	⊕ ⊕ ○ ○ Low	IMPORTANT
**TB**												
6	randomised trials	serious^c^	very serious^f^	not serious	not serious	none	520	280	-	SMD **1.27 SD lower** (2.67 lower to 0.14 higher)	⊕ ○ ○ ○ Very low	IMPORTANT
**TC**												
5	randomised trials	serious^c^	very serious^g^	not serious	not serious	none	412	227	-	SMD **0.71 SD lower** (1.38 lower to 0.04 lower)	⊕ ○ ○ ○ Very low	IMPORTANT
**TG**												
4	randomised trials	serious^c^	very serious^h^	not serious	not serious	none	362	177	-	SMD **1.33 SD lower** (2.75 lower to 0.08 higher)	⊕ ○ ○ ○ Very low	NOT IMPORTANT
**LDL**												
4	randomised trials	serious^c^	very serious^i^	not serious	not serious	none	362	177	-	SMD **1 SD lower** (2.08 lower to 0.07 higher)	⊕ ○ ○ ○ Very low	NOT IMPORTANT
**HDL**												
3	randomised trials	not serious	very serious^j^	not serious	not serious	none	184	90	-	SMD **0.29 SD higher** (0.71 lower to 1.29 higher)	⊕ ⊕ ○ ○ Low	NOT IMPORTANT
**Overall adverse reactions**												
6	randomised trials	not serious	not serious	not serious	not serious	none	423/521 (81.2%)	232/280 (82.9%)	**RR 0.99** (0.92–1.05)	**8 fewer per 1,000** (from 66 fewer to 41 more)	⊕ ⊕ ⊕ ⊕ High	IMPORTANT
**Serious adverse reactions**												
6	randomised trials	not serious	not serious	not serious	not serious	none	43/521 (8.3%)	25/280 (8.9%)	**RR 1.10** (0.71–1.72)	**9 more per 1,000** (from 26 fewer to 64 more)	⊕ ⊕ ⊕ ⊕ High	IMPORTANT
**Pruritus**												
6	randomised trials	not serious	not serious	not serious	not serious	none	137/519 (26.4%)	82/283 (29.0%)	**RR 0.87** (0.72–1.05)	**38 fewer per 1,000** (from 81 fewer to 14 more)	⊕ ⊕ ⊕ ⊕ High	IMPORTANT

**CI,** confidence interval; **MD,** mean difference; **RR,** risk ratio; **SMD,** standardised mean difference.

Explanations.

^a^ . I ^ 2= 76%.

^b^ . I ^ 2 = 75%.

^c^ . Sensitivity analysis suggested that the result was not robust.

^d^ . I ^ 2 = 62%.

^e^ . I ^ 2 = 89%.

^f^ . I ^ 2 = 98%.

^g^ . I ^ 2 = 92%.

^h^ . I ^ 2 = 97%.

^i^ . I ^ 2 = 96%.

^j^ . I ^ 2 = 90%.

### 3.4 Meta-analysis

#### 3.4.1 Biochemical response

**FIGURE 3:**
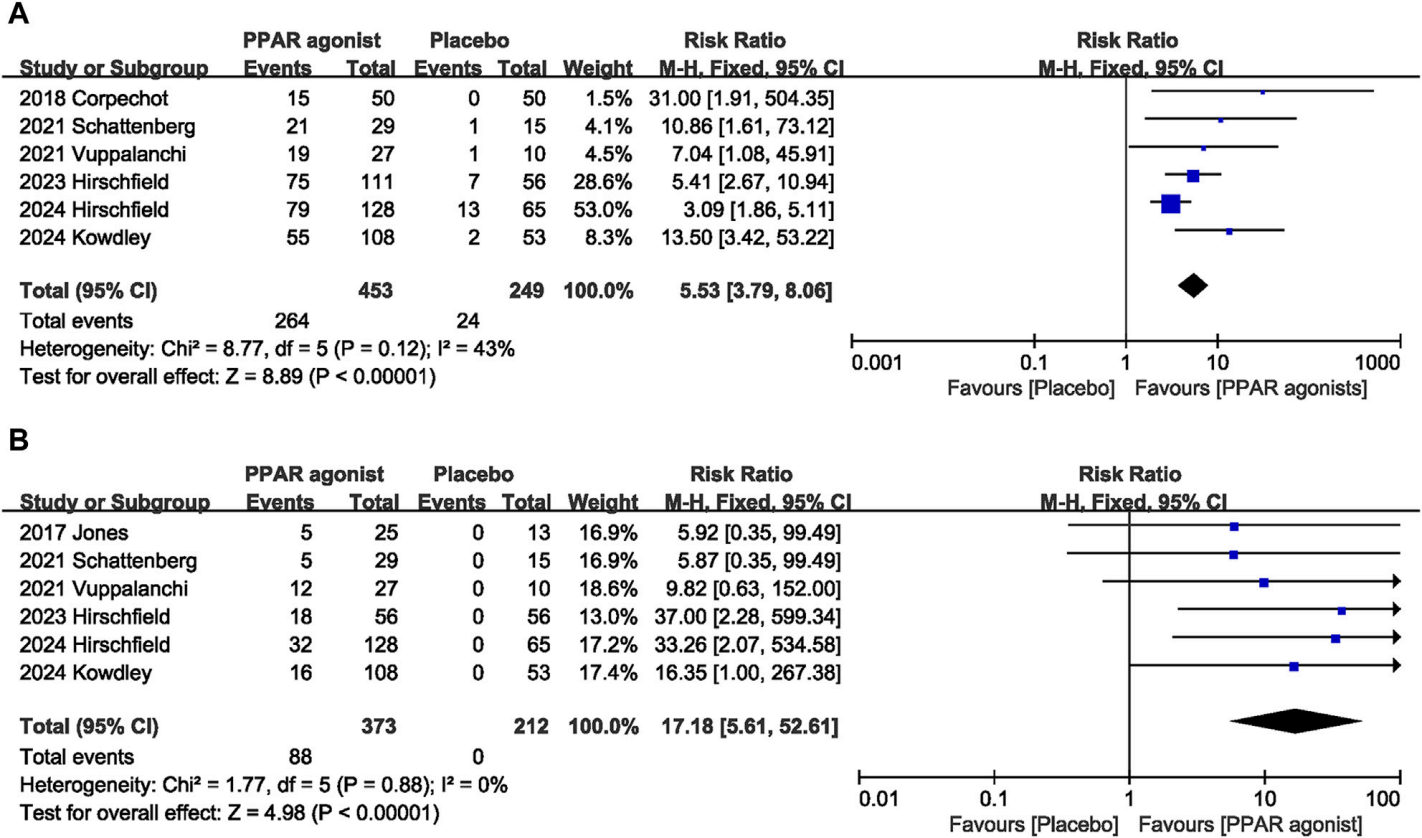
Effects of PPAR agonists on **(A)** biochemical response and **(B)** normalization of the ALP level.

Six studies (Corpechot et al., 2018; Schattenberg et al., 2021; Vuppalanchi et al., 2022; Hirschfield et al., 2023; Kowdley et al., 2024; Hirschfield et al., 2024) evaluated the effect of PPAR agonists on the biochemical response rate. Supplementation with PPAR agonists increased the biochemical response rate compared to the control group, but the difference was not statistically significant (RR: 5.53; 95% CI: 3.79, 8.06) (Figure 3A). There was no significant heterogeneity among studies (I^2^ = 43%, *p* = 0.12) (Table 3).

#### 3.4.2 Normalization of the ALP level

The combined effect of six datasets (Jones et al., 2017; Schattenberg et al., 2021; Vuppalanchi et al., 2022; Hirschfield et al., 2023; Kowdley et al., 2024; Hirschfield et al., 2024) showed that supplementation with PPAR agonists significantly increased the incidence of normalization of the ALP level (RR: 17.18; 95% CI: 5.61, 52.61), with no significant heterogeneity observed among studies (I^2^ = 0%, *p* = 0.88) (Figure 3B).

#### 3.4.3 ALT

**FIGURE 4:**
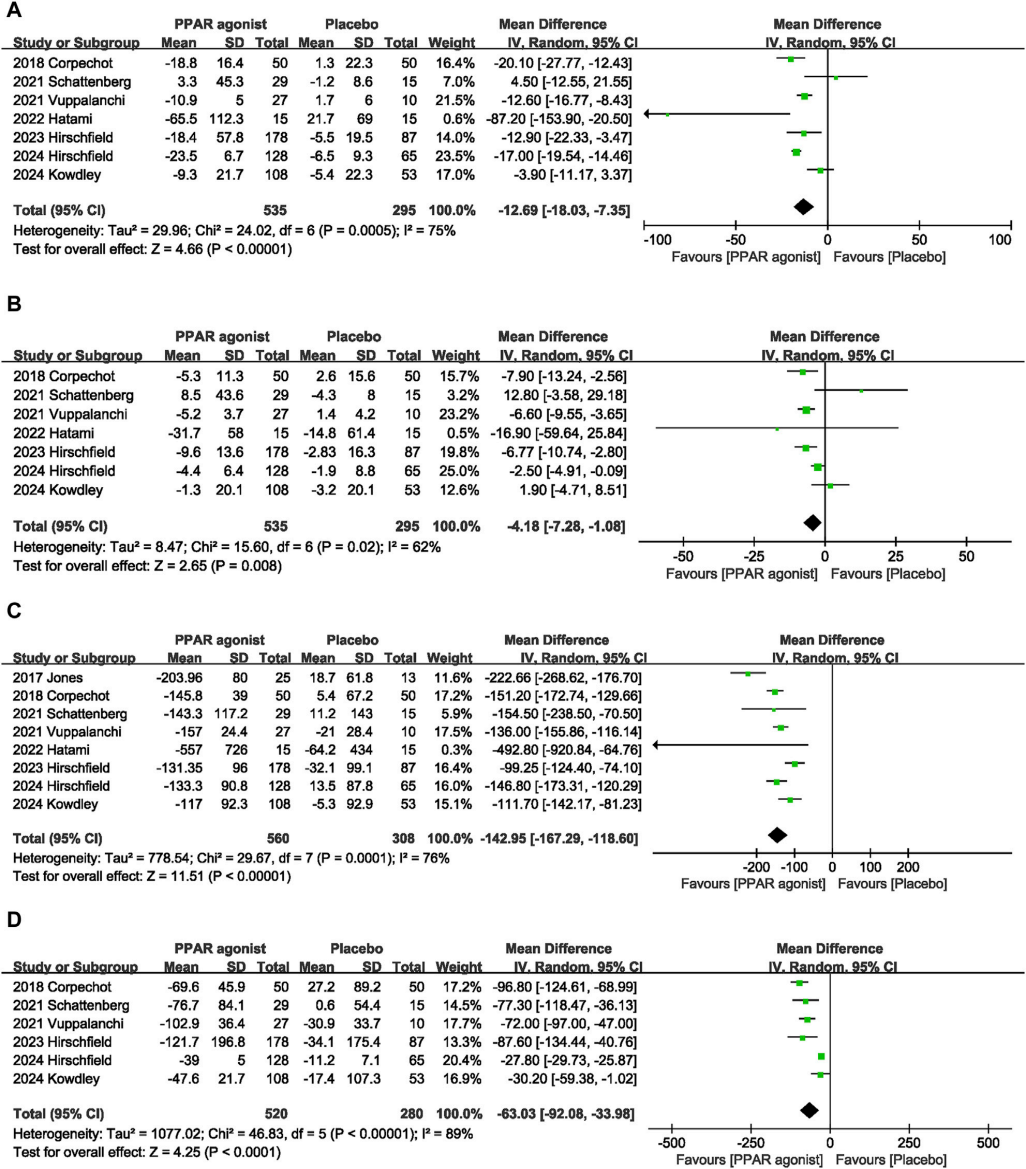
Effects of PPAR agonists on **(A)** ALT, **(B)** AST, **(C)** ALP, and **(D)** GGT.

Seven datasets (Corpechot et al., 2018; Schattenberg et al., 2021; Hatami et al., 2022; Vuppalanchi et al., 2022; Hirschfield et al., 2023; Kowdley et al., 2024; Hirschfield et al., 2024) evaluated the effect of PPAR agonists on ALT levels among patients. Compared with the placebo, significantly decreased the ALT level (MD: −12.69 U/L; 95% CI: −18.03, −7.35), and there was significant heterogeneity among studies (I^2^ = 75%, *p* = 0.0005) (Figure 4A).

#### 3.4.4 AST

A meta-analysis of seven trials (Corpechot et al., 2018; Schattenberg et al., 2021; Hatami et al., 2022; Vuppalanchi et al., 2022; Hirschfield et al., 2023; Kowdley et al., 2024; Hirschfield et al., 2024) indicated that PPAR agonists significantly reduced the AST level, relative to those in the placebo group (MD: −4.18 U/L; 95% CI: −7.28, −1.08). There was significant heterogeneity among these studies (I^2^ = 62%, *p* = 0.02) (Figure 4B).

#### 3.4.5 ALP

The pooled effect size of eight trials (Jones et al., 2017; Corpechot et al., 2018; Schattenberg et al., 2021; Hatami et al., 2022; Vuppalanchi et al., 2022; Hirschfield et al., 2023; Kowdley et al., 2024; Hirschfield et al., 2024) showed a significant reduction in the ALP level (MD: −142.95 U/L; 95% CI: −167.29, −118.60) in patients with PPAR agonists supplementation, relative to that of the control group. Furthermore, the heterogeneity was high (I^2^ = 76%, *p* = 0.0001) (Figure 4C).

#### 3.4.6 GGT

Six studies (Corpechot et al., 2018; Schattenberg et al., 2021; Vuppalanchi et al., 2022; Hirschfield et al., 2023; Kowdley et al., 2024; Hirschfield et al., 2024) reported data on GGT, and pooled evaluation of the six trials showed that supplementation with PPAR agonists significantly improved GGT levels (MD: −63.03 U/L; 95% CI: −92.08, −33.98) (Figure 4D).

#### 3.4.7 TB

**FIGURE 5:**
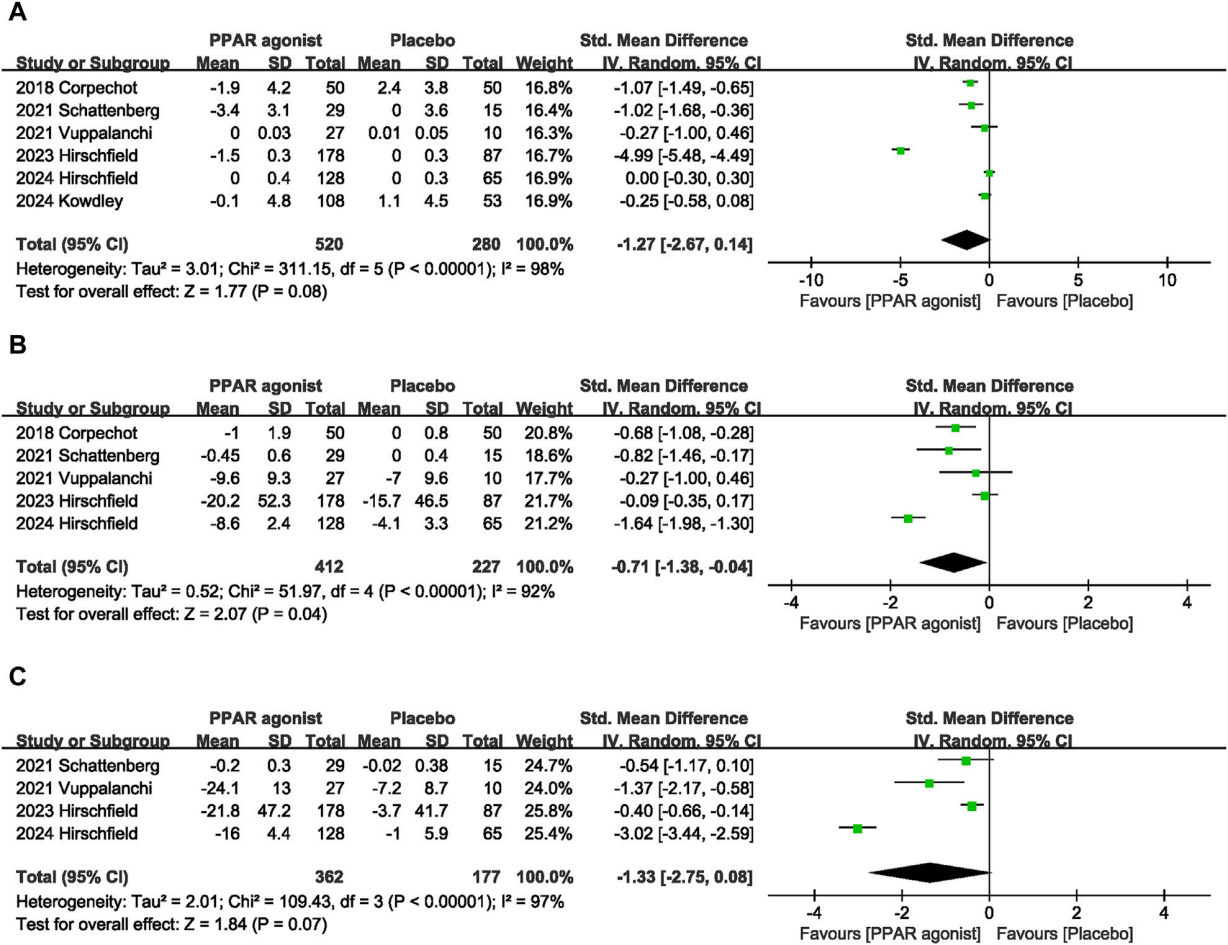
Effects of PPAR agonists on **(A)** TB, **(B)** TC, and **(C)** TG.

Pooled data from six studies (Corpechot et al., 2018; Schattenberg et al., 2021; Vuppalanchi et al., 2022; Hirschfield et al., 2023; Kowdley et al., 2024; Hirschfield et al., 2024) showed that PPAR agonists reduced TB levels, but there was no statistical difference (SMD: −1.27; 95% CI: −2.67, 0.14) (Figure 5A).

#### 3.4.8 TC

Five studies (Corpechot et al., 2018; Schattenberg et al., 2021; Vuppalanchi et al., 2022; Hirschfield et al., 2023; Hirschfield et al., 2024) provided data on TC. Compared with the control group, PPAR agonists were associated with a reduction in the TC (SMD: −0.71; 95% CI: −1.38, −0.04) (Figure 5B).

#### 3.4.9 TG

The pooled effect size of four trials (Schattenberg et al., 2021; Vuppalanchi et al., 2022; Hirschfield et al., 2023; Hirschfield et al., 2024) indicated no significant difference in TG levels between patients supplemented with PPAR agonists and the placebo group (SMD: −1.33; 95% CI: −2.75, 0.08) (Figure 5C).

#### 3.4.10 LDL

**FIGURE 6:**
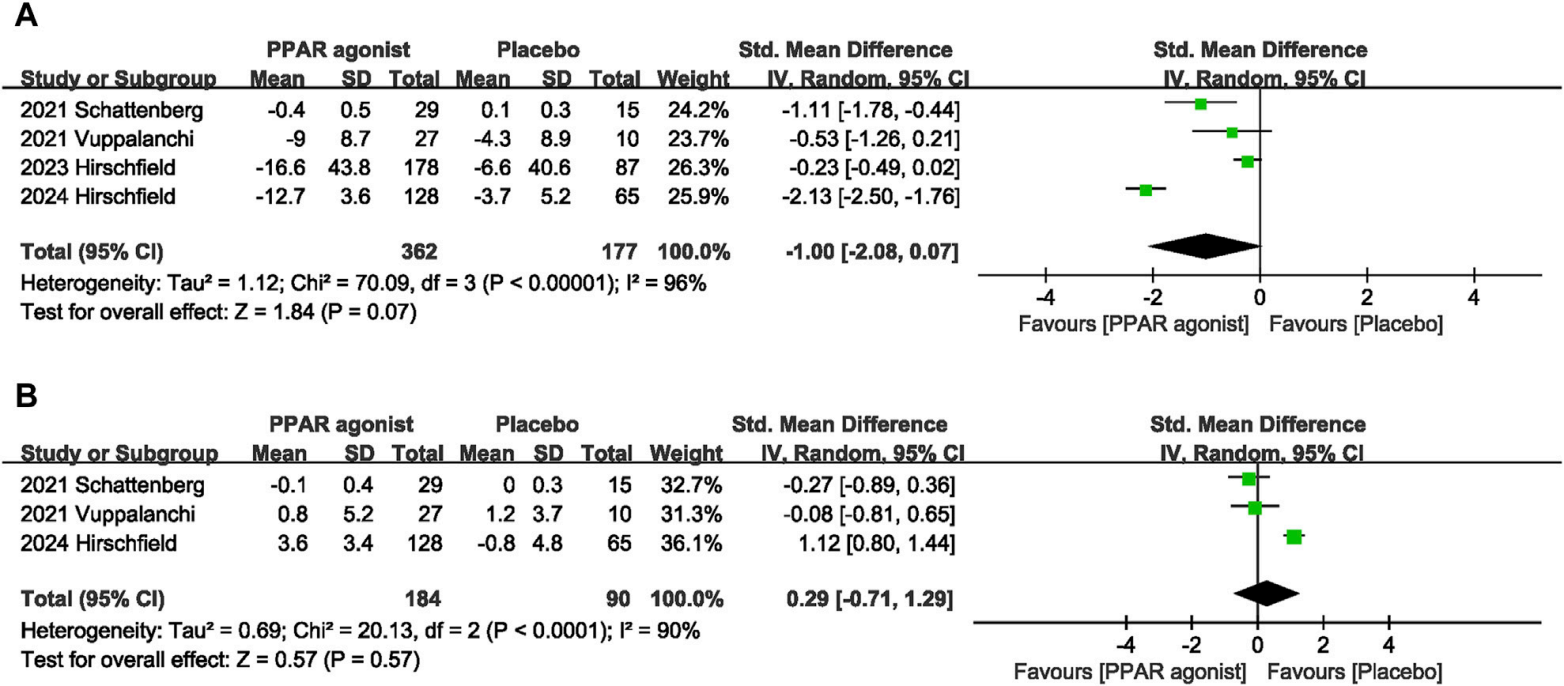
Effects of PPAR agonists on **(A)** LDL and **(B)** HDL.

The impact of PPAR agonists on LDL was evaluated in 4 studies (Schattenberg et al., 2021; Vuppalanchi et al., 2022; Hirschfield et al., 2023; Hirschfield et al., 2024). PPAR agonists did not improve LDL levels (SMD: −1.00; 95% CI: −2.08, 0.07) (Figure 6A).

#### 3.4.11 HDL

Three studies (Schattenberg et al., 2021; Vuppalanchi et al., 2022; Hirschfield et al., 2024) reported data on HDL, and pooled evaluation of the three trials showed no significant effect of PPAR agonists supplementation on the level of HDL (SMD: 0.29; 95% CI: −0.71, 1.29) (Figure 6B).

#### 3.4.12 Adverse effects

**FIGURE 7:**
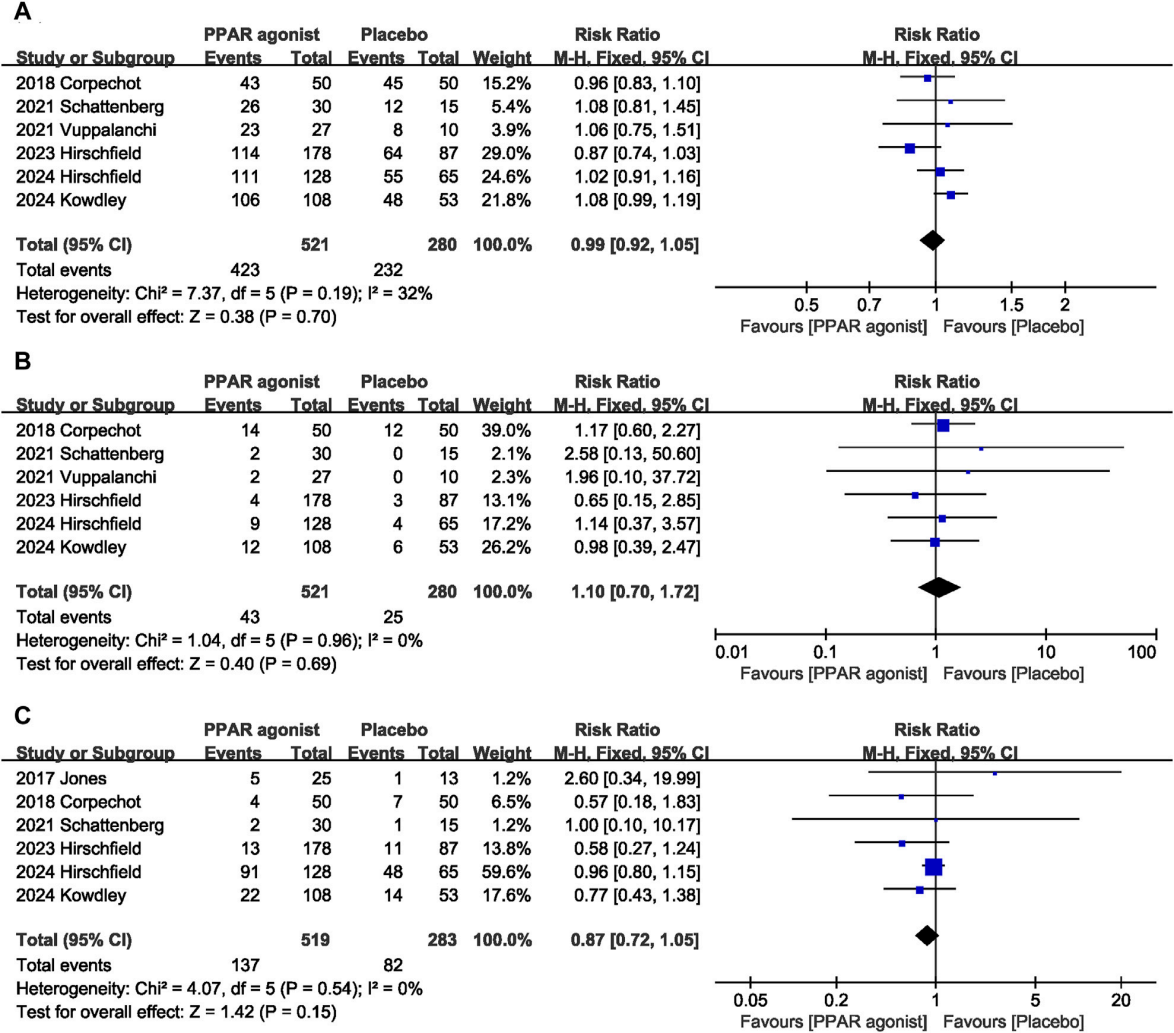
Effects of PPAR agonists on **(A)** overall adverse reactions, **(B)** serious adverse reactions, and **(C)** pruritus.

Adverse effects were assessed in seven studies (Jones et al., 2017; Corpechot et al., 2018; Schattenberg et al., 2021; Vuppalanchi et al., 2022; Hirschfield et al., 2023; Kowdley et al., 2024; Hirschfield et al., 2024). PPAR agonists did not increase the incidence of overall adverse reactions (RR: 0.99; 95% CI: 0.92, 1.05) (Figure 7A), serious adverse reactions (RR: 1.10; 95% CI: 0.70, 1.72) (Figure 7B), or pruritus (RR: 0.87; 95% CI: 0.72, 1.05) (Figure 7C).

### 3.5 Sensitivity analysis

Sensitivity analysis showed that no single study affected the overall effect size of the biochemical response, normalization of the ALP level, overall adverse reactions, serious adverse reactions, pruritus, ALP, ALT, HDL, and GGT. The size of the pooled effect of AST was influenced by Vuppalanchi et al. (2022) (MD: −3.34 U/L; 95% CI: −7.20, 0.52) or Hirschfield et al. (2023a) (MD: −3.34 U/L; 95% CI: −7.16, 0.29). The overall effect size of TB changed when the study by Hirschfield et al. (2023a) (SMD, −0.50; 95% CI, −0.94, −0.05, *p* = 0.03) was excluded. The size of the pooled effect of the TC was influenced by Corpechot et al. (2018b) (SMD, −0.71; 95% CI, −1.60, 0.18) and 2021 Schattenberg et al. (Schattenberg et al., 2021) (SMD, −0.68; 95% CI, −1.48, 0.11).The overall effect size for the TG changed when the study by Hirschfield et al. (2023a) (SMD, −1.66; 95% CI, −3.30, −0.01) or Kowdley et al. (2024b) (SMD, −0.66; 95% CI, −1.17, −0.16) was excluded. The overall effect size for the LDL changed when the study by Hirschfield et al. (2023a) (SMD, −1.29; 95% CI, −2.30, −0.28) or Hirschfield et al. (2024) (SMD, −0.56; 95% CI, −1.10, −0.02) was excluded.

## 4 Discussion

This is the first meta-analysis to assess the efficacy of PPAR agonists for the management of PBC. Our meta-analysis, based on evidence from high-quality RCTs, indicated that that the PPAR agonists can effectively increase the rate of biochemical response and normalization of the ALP level. In addition, PPAR agonists demonstrated superior efficacy in improving the level of ALP, ALT, AST, GGT, and TC compared with that using placebo. In terms of safety, the incidence of adverse effects of PPAR agonists is similar to that of placebo.

Biochemical response is a key predictor of clinical prognosis for PBC and insufficient biochemical response after UDCA treatment is strongly associated with the risk of cirrhosis progression and death (Lin et al., 2024). In addition, Harms et al. (2018) found that biochemical non-response to UDCA significantly increased the risk of cirrhosis related complications such as ascites, variceal bleeding, and hepatic encephalopathy in PBC patients (HR: 5.52:4.17–7.33). Therefore, improving the biochemical response rate is particularly important for the treatment of PBC. Our results show that PPAR agonists can effectively improve the biochemical response rate, which has important clinical value, because the increase of biochemical response rate may help improve the clinical outcome of PBC. Therefore, future RCT studies are warranted to further evaluate the impact of PPAR agonists on long-term outcomes in patients with PBC. ALP is an important indicator of the diagnosis and prognosis of PBC and is considered an alternative endpoint for therapeutic clinical trials (Xu et al., 2022; Lin et al., 2024). Among PBC patients with an ALP≤ 2.0x ULN, the 10-year survival rate was 84%, while the survival rate was only 62% for patients with an ALP greater than 2.0x ULN (*p* < 0.0001) (Lammers et al., 2014). Normalization of the ALP level was associated with the lowest risk of liver transplantation or death in patients with PBC (Murillo Perez et al., 2020; Kowdley et al., 2024). Murillo Perez et al. (2020) evaluated the prognostic significance of ALP level 1 year after treatment and found that the 10-year survival rate was 93.2% in patients with ALP≤1 × ULN and 86.1% in patients with ALP 1.0–1.67 × ULN. Our results suggest that PPAR agonists therapy effectively reduces ALP levels (MD: –142.95 U/L). In addition, 23.6 percent of patients achieved normalization of the ALP level after treatment, while no patients in placebo normalized ALP levels. This is similar to the results of some previous studies (Li et al., 2022; Liu et al., 2023). In addition, the retrospective study by Ghonem et al. (2020) also observed the benefit of PPAR agonists on ALP.

ALT, AST, TB and GGT were related biochemical indexes of PBC activity (Hirschfield et al., 2023). Our meta-analysis suggests that PPAR agonists is effective in reducing ALT, AST, and GGT levels. Although TB levels decreased after treatment, there was no statistical difference between the PPAR agonists group and the placebo group. However, this result is based on data from a limited number of studies, and more research is needed to further clarify the benefits of PPAR agonists on TB. Dyslipidemia is also common in patients with PBC. The PPAR agonist seladelpar was originally developed to lower blood lipids in patients with mixed dyslipidemia (Jones et al., 2017). Our study found that after PBC patients received PPAR agonists treatment, serum TC levels decreased and TG and LDL and HDL levels remained stable.

The benefits of PPAR agonists on PBC may be related to the following mechanisms. PPAR consists of three isomers: α, β/δ and γ. PPARα is involved in the regulation of homeostasis of cholesterol and bile acids. Multidrug resistant protein 3 (MDR3) plays a key role in bile salt secretion, and PPARα can reduce bile acid synthesis by increasing MDR3 expression (Hatami et al., 2022). In addition, 7α-hydroxylase (CYP7A1) is a key enzyme in the classical pathway of bile acid synthesis, which catalyzes the hydroxylation of cholesterol at site 7, while the activation of PPARa and PPARδ downregulates the expression of CYP7A1 (Jones et al., 2017; Schattenberg et al., 2021). Then, PPARa and the PPARδ agonist elafibranor may also play a beneficial role by increasing bile acid output and forming non-toxic bile acid micelles in the bile duct (Schattenberg et al., 2021). Furthermore, some studies have found that the activation of PPARδ can induce the anti-inflammatory effects of macrophages, reduce liver inflammation, and improve liver fibrosis (Odegaard et al., 2008; Haczeyni et al., 2017; Hirschfield et al., 2023).

Safety is an important factor affecting the further application of drugs. Our study showed no significant difference in the incidence of adverse reactions or serious adverse reactions between the PPAR agonists group and the placebo group. In addition, taking into account the effect of pruritus, we separately assessed the incidence of pruritus during treatment. The results showed that the pruritus rate in the PPAR agonists group was lower than that in the placebo group, but there was no statistical difference. Similarly, Kremer et al. (2022) found that in patients with PBC accompanied by moderate to severe pruritus, administration of 5 mg or 10 mg of seladelpar significantly improved pruritus symptoms. This finding is in contrast to the second-line treatment for PBC, obeticholic acid, which has been shown to exacerbate pruritus (Kowdley et al., 2024). Therefore, these results suggest that PPAR agonists is well tolerated.

Our study has the following strengths, on the one hand, we conducted a comprehensive literature search, developed strict inclusion criteria, and included only RCTs. In addition, the included studies were all high-quality randomized, double-blind, placebo-controlled trials with high and extensive reference value. On the other hand, sensitivity analysis suggested that the primary outcome of our study was robust, which further enhanced the reliability of the study conclusions.

Despite these strengths, our study has the following limitations. First, the number of included studies was limited, and some studies were small sample size RCTs, which may limit the statistical power and the reliability of the conclusions drawn from those studies. Second, for the selected studies, diversity in study design, population, and intervention protocols may introduce variability, with high heterogeneity in some outcomes. However, due to the limited number of included studies, subgroup analysis was not possible. In addition, There is always a risk of publication bias in meta-analyses, as studies with positive results are more likely to be published than those with negative or inconclusive findings. However, due to the limited number of studies (<10), it is impossible to further use a funnel diagram to evaluate potential publication bias. Finally, data to assess liver histological changes and long-term prognosis were lacking in the included studies. Considering the benefits of PPAR agonists on liver biochemical markers, further RCTs to assess the effects of PPAR agonists on long-term prognosis and histological changes in patients with PBC are warranted.

In conclusion, the results of our meta-analysis suggest that in patients with PBC, PPAR agonists are safe, well-tolerated, and associated with improvements in liver-associated biomarkers and multiple metabolic parameters. PPAR agonists have the potential to be an attractive second-line strategy for the future treatment of PBC, and high-quality clinical trials with longer follow-up are needed to validate the benefits of PPAR agonists for PBC.
